# Resident Appointments and the Present Emergency

**Published:** 1915-02-20

**Authors:** 


### The Hospital

The Workers' Newspaper of Administrative Medicine and Institutional Life, Administration, National Insurance and Health.

No. 1496, Vol. LVII. SATURDAY, FEBRUARY 20, 1915. PRICE ONE PENNY.

### RESIDENT APPOINTMENTS AND THE PRESENT EMERGENCY.

The difficulty of obtaining house physicians and house surgeons for the hospitals is, as we ventured to anticipate, becoming more and more pressing, and we now have a very plain intimation that unless the position can be redeemed the hospital facilities available for the general population may have to be reduced by the closure of wards and of out-patient departments. In some quarters criticisms have been advanced on the increased salaries expected by candidates for these positions, and it has been suggested that such expectations are the fruit of an imperfect patriotism. The answer is that the present state of affairs is far from being due entirely to the war. No doubt the war has made it more acute by taking away from civil practice a large number of the junior members of the profession. But even prior to last August the hospitals had found that candidates were scarce and that the acute competition of former days had disappeared. For this, the large number of appointments available to the younger men and created by various Acts of Parliament was responsible. The demand had increased while the supply had remained stationary, and inevitably, therefore, by stern economic law, salaries had to be increased. For many years the hospitals enjoyed the advantages of a supply greater than the demand, and had accordingly been able to secure service for very modest remuneration. Now that the position is reversed, it is in the very nature of things that this remuneration must be increased. The general market price has been raised and the hospitals may not hope to escape the consequence. The same course of reasoning is applicable to the fees expected by *locum tenentes* who take temporary duty at hospitals. The figure is fixed by the interaction of supply and demand, and it is unreasonable to reproach these gentlemen for asking the sums which they can readily obtain elsewhere. Certainly there is just now a reasonable claim for moderation and forbearance and sacrifice. But this claim applies to every citizen, and is not to be concentrated in some special measure on the medical profession. It is not to be expected that young men with the battle of life in front of them shall throw away the advantages of an economic position which has grown naturally out of legislative and administrative action and is not the result of any artificial and arbitrary arrangement or restraint. The truth is that these hospital appointments have become relatively less attractive than they were, and to that position hospital managers must adjust themselves as best they may.

The immediate influence of the war in this matter has been not so much to increase salaries as to abolish candidates. The increase was well within sight before the war appeared, and, while the pace may have been hurried somewhat, the movement was inevitable in any event. The present emergency, therefore, resides not in the need for larger payments, but in the absence of available men. The demands of the war, arriving during a prevailing scarcity, have created a famine, and this is likely to increase rather than to diminish. In other words, the brief fact is that, whatever salaries are offered, there are more vacancies than candidates to fill them.

To meet the position created by these exceptional circumstances is a matter of great difficulty. But we desire to urge very strongly that to close wards or to shut up out-patient departments is the very last course which should be adopted. Fortunately, the winter, so far, has not been marked by excessive illness among the classes which look to the hospitals for succour. But the amount of hospital provision for the civil population has been diminished by the needs of the sick and wounded soldiers, and we have yet some two months of possible bad weather in front of us. Whatever may be true elsewhere, London, at all events, cannot afford further to lessen its hospital facilities. There is only one alternative, and that is to call upon the goodwill and generosity of individual members of the profession for increased service. Not every man may stand in the fighting line, and not every doctor may serve with the Expeditionary Force. But many, if not all, of those excluded from these activities may, by a readiness to make some personal sacrifice of time, of ambition, or of some cherished plan, render help to keep our hospitals open and active. Is not this necessary work worthy of an occasion which calls all men of goodwill to yield personal interests to the general welfare?

An invitation has been issued, at least by one hospital, for volunteers from the ranks of civil practitioners. Let other institutions which find themselves in difficulties follow this example, and we cannot doubt of the response. At present the urgency is not widely known, and unless by such an appeal as is here suggested it will not be known; practitioners can hardly offer themselves until they are asked. In all this a great responsibility rests on the honorary medical staffs. Their sympathy and practical co-operation are, of course, essential, and they will, we are sure, recognise that the present is no occasion for punctiliousness or formality. Let all honest help be welcomed with cordiality and with a determination to arrange a practical scheme in which, by division of labour, the hospital organisation can be continued with success. The young men fail because they are called elsewhere by the claims of duty. But our hospitals must not fail: nor need they, if their wants are made known and if all concerned work together.

